# Enhancer-derived long non-coding RNAs *CCAT1* and *CCAT2* at rs6983267 has limited predictability for early stage colorectal carcinoma metastasis

**DOI:** 10.1038/s41598-020-79906-7

**Published:** 2021-01-11

**Authors:** Lai Fun Thean, Christopher Blöcker, Hui Hua Li, Michelle Lo, Michelle Wong, Choong Leong Tang, Emile K. W. Tan, Steven G. Rozen, Peh Yean Cheah

**Affiliations:** 1 Department of Colorectal Surgery, Singapore General Hospital, Academia, Level 9, Discovery Tower, 20 College Road, Singapore, 169856 Singapore; 2 Department of Physics, Umeå University, 90187 Umeå, Sweden; 3 Health Service Research Unit, Singapore General Hospital, Singapore, Singapore; 4 Duke-NUS Center for Computational Biology, Duke-NUS Medical School, National University of Singapore, Singapore, Singapore; 5 Saw Swee Hock School of Public Health, National University of Singapore, Singapore, Singapore; 6 Duke-NUS Medical School, National University of Singapore, Singapore, Singapore

**Keywords:** Cancer, Computational biology and bioinformatics, Genetics, Biomarkers, Oncology

## Abstract

Up-regulation of long non-coding RNAs (lncRNAs), colon-cancer associated transcript (*CCAT*) *1* and *2*, was associated with worse prognosis in colorectal cancer (CRC). Nevertheless, their role in predicting metastasis in early-stage CRC is unclear. We measured the expression of *CCAT1*, *CCAT2* and their oncotarget, *c-Myc*, in 150 matched mucosa-tumour samples of early-stage microsatellite-stable Chinese CRC patients with definitive metastasis status by multiplex real-time RT-PCR assay. Expression of *CCAT1*, *CCAT2* and *c-Myc* were significantly up-regulated in the tumours compared to matched mucosa (*p* < 0.0001). The expression of *c-Myc* in the tumours was significantly correlated to time to metastasis [hazard ratio = 1.47 (1.10–1.97)] and the risk genotype (GG) of rs6983267, located within *CCAT2*. Expression of *c-Myc* and *CCAT2* in the tumour were also significantly up-regulated in metastasis-positive compared to metastasis-negative patients (*p* = 0.009 and *p* = 0.04 respectively). Nevertheless, integrating the expression of *CCAT1* and *CCAT2* by the Random Forest classifier did not improve the predictive values of ColoMet19, the mRNA-based predictor for metastasis previously developed on the same series of tumours. The role of these two lncRNAs is probably mitigated via their oncotarget, *c-Myc*, which was not ranked high enough previously to be included in ColoMet19.

## Introduction

Colorectal Cancer (CRC) is the third highest incidence cancer and a leading cause of cancer mortality worldwide, attributable mainly to metastasis to distal organs^1^. Early stage (Stage I and II) CRC patients, whose cancers are confined to the colonic wall, are considered curative by surgery alone. However, up to 25% of these patients still succumb to metastasis within 5 years^2^. It is thus imperative that an accurate diagnostic tool be developed that can identify metastasis-prone early stage patients that may benefit from adjuvant therapy and spared the rest of the patients from unnecessary and toxic therapy.

We have previously identified an expression–based metastasis predictor, ColoMet19, in early-stage CRC^3,4^. We have also shown that mutation status of 20 frequently mutated genes and expressions of 2549 miRNAs profiled on the same design set did not improve the predictor^4^. The final predictor has a positive predictive value (PPV) and negative predictive value (NPV) of 0.67 and 0.86 respectively indicating that early-stage CRC patients who tested positive have a 67% risk of developing metastases and conversely those who tested negative have 86% probability of remaining metastasis-free. Though ColoMet19 has clinical utility, we aimed to integrate additional features to improve its PPV to lend higher confidence for clinical translation.

Long non-coding RNA (lncRNA) has recently been implicated in CRC progression and survival. Colon cancer associated transcript 1 (*CCAT1*) and *CCAT2* are two enhancer-derived lncRNAs located about 500 and 300 kb respectively upstream of their target *c-Myc* at chromosome 8q24^5–8^. The first CEU-identified single nucleotide polymorphism (SNP) associated with CRC risk, rs6983267, is located within the lncRNA *CCAT2* (Fig. S1). Previous studies have reported that this SNP is in an enhancer region that could regulate *c-Myc*, an oncoprotrein in the Wnt signaling pathway^9,10^. We have also shown that this SNP was associated with sporadic CRC risk in Singapore Chinese population^11^. Enhancer-derived lncRNAs were reported to be stable non-coding RNAs that modify the chromatin by binding to CTCF-marked topologically associating domains (TADs) thus altering genome architecture. Such cis-acting lncRNA-mediated chromosomal looping could be another mechanism affecting distal targets^12,13^. Accumulating evidence thus suggests that *CCAT1* and *CCAT2* are two promising enhancer-derived lncRNAs that could serve as disease biomarkers^7,14,15^. Nevertheless, their role in metastasis prediction was hitherto unclear*.*

In this study, we aimed to investigate whether the expression of *CCAT1* and *CCAT2* was coordinately upregulated in the same series of tumours and whether their up-regulation correlated with that of their target *c-Myc* in Singapore Chinese patients. Further, we intended to determine whether the G risk allele of the rs6983267 SNP upregulates the expression of these lncRNAs and their oncotarget *c-Myc* in the tumours compared to the T allele. More importantly, we aimed to explore whether the expression of these lncRNAs improve the metastasis predictive values of ColoMet19.

## Results

Three metastasis-negative samples were excluded either because of poor RNA integrity or the expression of *CCAT2* in the matched mucosa samples was below the limits of detection after repeated attempts. Four metastasis-positive samples were excluded due to recent new findings which throw doubt on their status. Thus the analysis was performed on 143 (46 metastasis-positive and 97 metastasis-negative) samples.

### Relative expression of *CCAT1*, *CCAT2* and *c-Myc*

**Figure 1:**
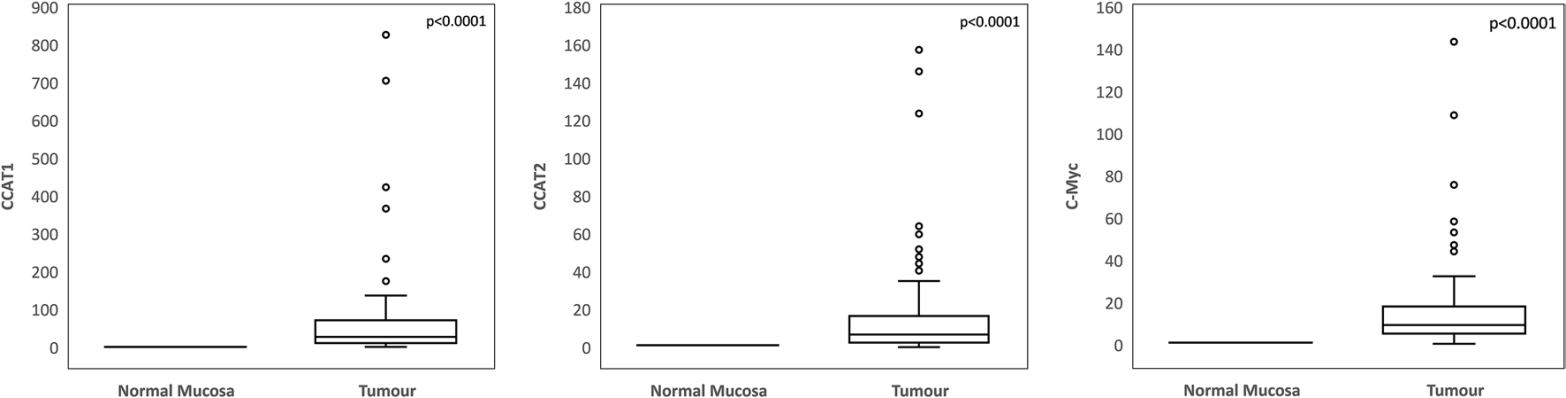
Boxplots of *CCAT1*, *CCAT2* and *c-MYC* expression between matched mucosa and tumours.

The relative quantitation of *CCAT1*, *CCAT2* and *C-Myc* was investigated. Box plot showed that the expression of *CCAT1*, *CCAT2* and *C-Myc* in the tumours was significantly (up to hundreds-fold) up-regulated in the tumours compared to their matched mucosa (*p* < 0.0001, Fig. 1).

### Expression of *c-Myc* was significantly correlated to time to metastasis

The expression of *c-Myc* in the tumours as well as the matched mucosa was significantly correlated to time to metastasis in this series (Table 1). Notably, the hazards ratio (HR) in the mucosa was in the opposite direction from that of tumour. *c-Myc* expression in the tumour (ΔCtT) was positively correlated (HR = 1.47) whilst that in the mucosa (ΔCtM) was inversely correlated to time to metastasis (HR = 0.68). Kaplan–Meier plot by *c-Myc* expression in the tumour (dichotomized into high and low using the mean expression value) indicates that *c-Myc* expression was significantly correlated to metastasis free survival (Fig. S2; log rank *p* = 0.004).

**Table 1:** Univariable analysis of time to metastasis by Cox regression.

	No of events	No of patients	HR (95% CI)	*p* value
CCAT1 ∆Ct T	46	142	1.11 (0.89, 1.40)	0.3536
CCAT1 ∆Ct M	46	142	0.88 (0.73, 1.06)	0.1883
CCAT1 ∆∆Ct	46	142	0.89 (0.77, 1.02)	0.1049
CCAT2 ∆Ct T	46	142	1.14 (0.97, 1.34)	0.1138
CCAT2 ∆Ct M	46	142	0.92 (0.79, 1.06)	0.2528
CCAT2 ∆∆Ct	46	142	0.90 (0.81, 1.00)	0.0496
C-MYC ∆Ct T	46	142	1.47 (1.10, 1.97)	**0.0107**
C-MYC ∆Ct M	46	142	0.68 (0.47, 0.97)	**0.0337**
C-MYC ∆∆Ct	46	142	0.72 (0.58, 0.88)	**0.0022**

Bold is significant (*p* < 0.05) value.

The expression of *CCAT1* and *CCAT2* in both the matched mucosa and the tumours were not significantly correlated to time to metastasis (Table 1).

The expression of *CCAT1* and *CCAT2* in the matched mucosa was at the limit of detection. Due to this low expression (and hence low reliability) and the opposing function of *c-Myc* in the tumours compared to the matched mucosa (Table 1), the expression of the three genes in the tumours normalized to endogenous control (β-actin), ΔCt T, was the expression used in further analysis.

### Expression of *CCAT2* and *c-Myc* was significantly correlated with metastasis status

**Figure 2:**
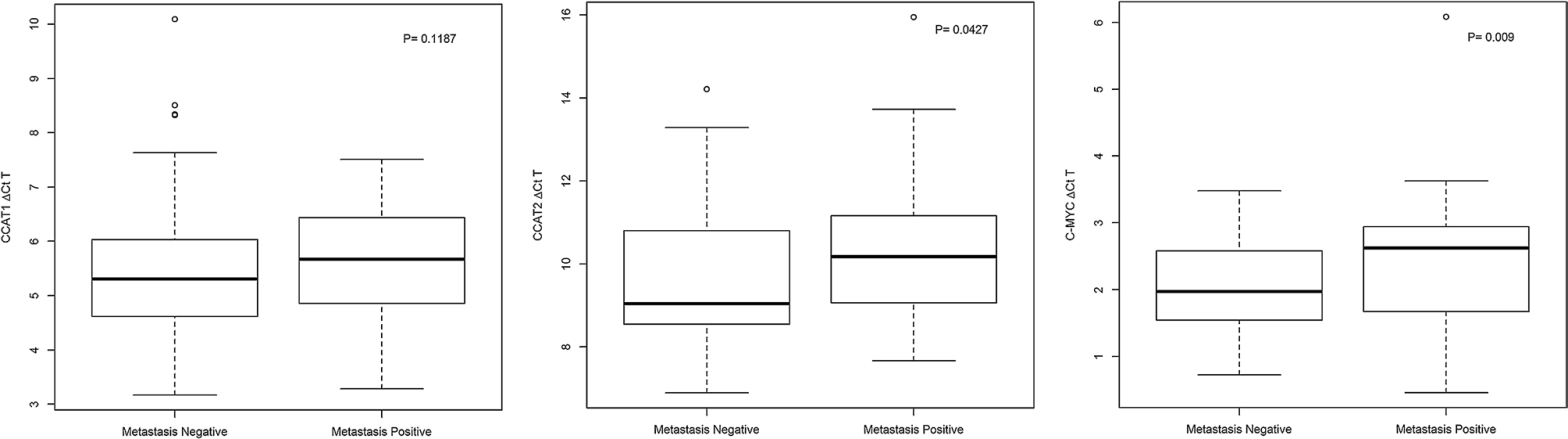
Boxplots of *CCAT1*, *CCAT2* and *c-MYC* expression between tumours of metastasis negative vs metastasis positive patients.

The expression of the lncRNA *CCAT2* and its oncotarget *c-Myc* was significantly higher in metastasis-positive patients compared to metastasis-negative patients. However, the expression of lncRNA *CCAT1* was not significantly different between the metastasis-positive and metastasis-negative patients by Mann–Whitney U test (Fig. 2).

### Integrating expression of *CCAT1* and *CCAT2* with ColoMet19

**Figure 3:**
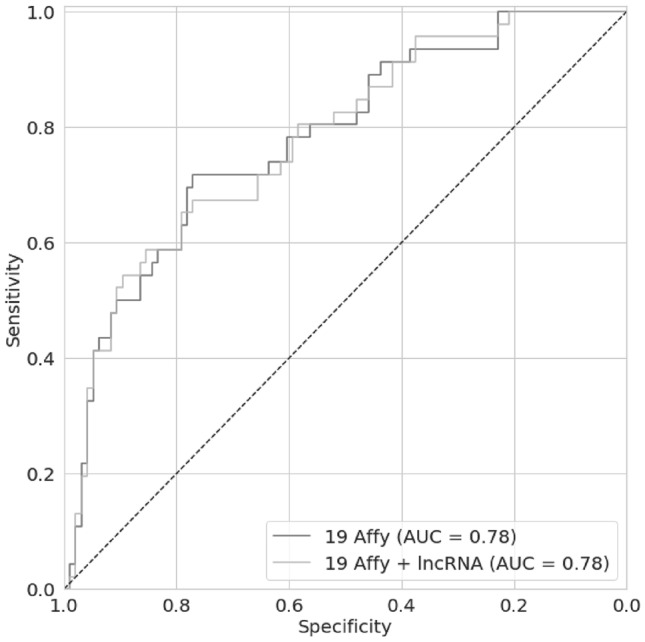
Receiver operating characteristic (ROC) curves for Random Forest feature selection. AUC, Area Under Curve.

We explored next whether the expression of the two lncRNAs can improve the predictive value of ColoMet19 for early stage CRC prone to metastasis. The receiver operating characteristic (ROC) plot indicates that integrating the expression of *CCAT1* and *CCAT2* with that of the expression of the 19 genes in ColoMet19 did not improve the predictive parameters of ColoMet19 (Fig. 3). The performance matrices (AUC, PPV and NPV) were nearly the same with or without the lncRNAs (0.78, 0.66 and 0.86 respectively).

We also explored the ranking of *CCAT1*, *CCAT2* and *c-Myc* compared to the 193 genes initially selected from the microarray platform (Fig. 2, Ref.^4^). *CCAT1*, *CCAT2*, and *c-Myc* ranked 195, 170 and 158 respectively. *c-Myc* was not amongst the initial 193 genes selected probably because the microarray platform (U133 plus 2) was 3′ enriched while the Taqman assay for *c-Myc* real time experiment in this study was at exon 2, the transcription activation domain at the N-terminus of *c-Myc*.

### *c-Myc* expression was significantly correlated to that of *CCAT1*, *CCAT2* and the GG risk genotype of rs6983267

**Figure 4:**
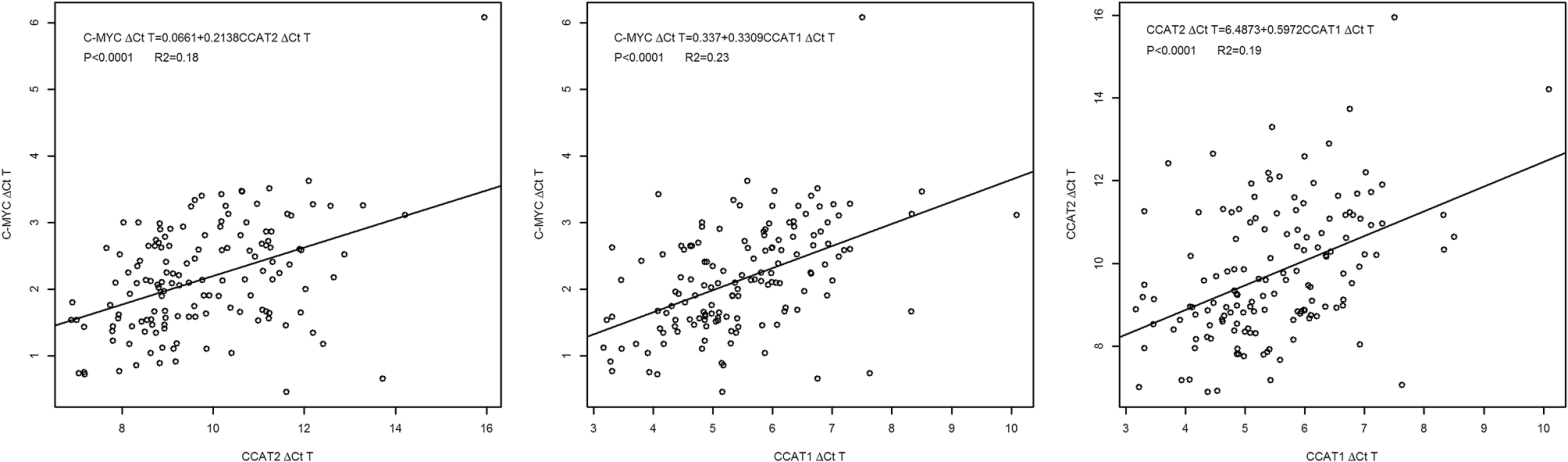
Linear regressions between *c-Myc*, *CCAT1*, and *CCAT2*.

*c-Myc* expression in the tumour was significantly correlated to that of *CCAT1* (R^2^ = 0.23, *p* < 0.0001) and *CCAT2* (R^2^ = 0.18, *p* < 0.0001) (Fig. 4). It was also significantly correlated to the risk genotype (GG) of the SNP rs6983267 (*p* = 0.0352, Table 2). The expression of lncRNA *CCAT2* also shows a trend of being higher in patients with GG genotype compared to that of GT/TT genotypes (Table 2).

**Table 2:** Expression levels in tumours of patients with GG compared to those with GT/TT genotypes at rs6983267.

	GG	GT/TT	*p* value
CCAT1 ∆Ct T	5.86 (5.38, 6.33)	5.42 (5.21, 5.63)	0.0969
CCAT2 ∆Ct T	10.32 (9.76, 10.87)	9.66 (9.36, 9.95)	0.0653
C-MYC ∆Ct T	2.47 (2.17, 2.77)	2.09 (1.95, 2.24)	**0.0352**

Bold is significant (*p* < 0.05) value.

## Discussion

In this study, we found the expression of *CCAT1*, *CCAT2* and *c-Myc* to be significantly up-regulated in the patients’ tumours compared to matched mucosa (Fig. 1). This is consistent with the findings of earlier reports from other populations although these previous studies used the expression of tumours normalized to internal control or a normal calibrator rather than matched mucosa^5–8^. We also found that *CCAT2* (but not *CCAT1*) expression was significantly higher in metastasis-positive patients compared to metastasis-negative patients suggesting that *CCAT2* may have some predictive value for metastasis (Fig. 2).

However, integrating the expression of these enhancer-derived lncRNAs with the 19 expressed genes in ColoMet19 did not increase the discriminative power of the metastasis signature (Fig. 3). This is in contrast to previous finding that these two lncRNAs have prognostic value in CRC^5–8^. One possible reason could be the differing end-points of the earlier studies and our study. The earlier studies used survival as end-point whilst in our study, definitive metastasis status was used. Metastasis status is a more direct endpoint than survival as it is documented clinical manifestation. Moreover, dichotomizing expression of markers (in this case, the lncRNAs) to perform survival analysis with log-rank *p* value has been shown recently to be associated with inherent inaccuracy^16^. Furthermore, the machine-learning classifier, Random Forest, adopted in this and an earlier study can rank the features by metastasis prediction capability. Integrating the expression of *CCAT1* and *CCAT2* did not improve the performance of ColoMet19; the PPV and NPV remained the same as previously reported when keeping the same voting threshold of 0.576^4^.

The oncotarget of these two lncRNAs, *c-Myc*, has slightly higher discriminative power than either of the lncRNAs (Table 1 and Fig. S2). Of the three genes investigated, only the expression of *c-Myc* in the tumour was significantly correlated to time to metastasis (Table 1). Of note, earlier studies have reported that *c-Myc* has conflicting apoptosis-induction and cell proliferation roles in normal and tumour tissues respectively^17,18^. Nevertheless, to our knowledge, this is the first time that *c-Myc* expression has been definitively shown to have opposing hazard ratio in normal (mucosa) vs cancerous (tumour) tissues (Table 1). This indicates that expression of a gene is both tissue and time-specific and caution has to be applied even when using matched tissue for normalization. Although the expression of *c-Myc* was significantly correlated to time to metastasis, it was ranked 158 in the 196 genes interrogated and hence also did not add discriminative value to the ColoMet19 signature. This is perhaps not surprising as current literature and software search engine (e.g. Clarivate analytics) did not rank *c-Myc* expression as informative for metastasis prediction for CRC^19^.

Only 23% and 18% of the variability in *c-Myc* expression in the tumours is attributable to the expression of *CCAT1* and *CCAT2* respectively (Fig. 4). The expression of *c-Myc* is reported to be influenced by the interplay of a platitude of proteins and lncRNAs other than *CCAT1* and *CCAT2*^20,21^. *CCAT1* (2628 nucleotide) is a much longer lncRNA than *CCAT2* (340 nucleotide) and previously reported to cause chromosomal looping via binding to CTCF to regulate *c-Myc*^12,22^. Its expression was up-regulated even more than *CCAT2* in the tumours compared to matched mucosa (Fig. 1) and account for a higher variability in *c-Myc* expression than *CCAT2* (Fig. 4). However, it was ranked lower than *CCAT2* as a metastasis-predicting feature suggesting that these parameters were not as informative for metastasis prediction in early stage CRC. Rather, *CCAT2* could have played a more important role than *CCAT1* via its physical interaction with TCF7L2 in the Wnt signaling pathway^6^. Though other lncRNAs have not been investigated, it is thus tempting to speculate that the role of lncRNAs in CRC metastasis prediction may be superseded by that of the target genes they regulate.

We showed that *c-Myc* expression was significantly up-regulated in patients with the GG risk genotype compared to the GT/TT genotypes at the rs6983267 SNP (Table 2), thus corroborating earlier observation that this -300 region could harbor a super enhancer regulating *c-Myc* in cis independent of the transcription of the lncRNA *CCAT2*^9,10,23^. The presence of the minor risk allele G was recently reported to be associated with worse prognosis of CRC through up-regulation of *c-Myc* transcription^24^. Of note, the GG genotype of rs6983267 appeared to have less of an effect on the transcription of *CCAT1* suggesting that the long range interaction with *c-Myc* is specific. The GG risk genotype also showed the trend of upregulating the transcription of the *CCAT2* locus within which the SNP resides, though this has not reached statistical significance.

We searched the GEO database for another CRC lncRNA expression dataset with metastasis information to verify the findings of this study. However, we could not find any, reiterating the difficulty of stratifying early-stage CRC patients by metastasis, and hence the uniqueness of our study. In conclusion, the expression of the two enhancer-derived lncRNAs *CCAT1* and *CCAT2* did not have additional discriminative power more than the 19 expressed genes in ColoMet19 for metastasis prediction in early stage microsatellite-stable sporadic CRC. Their contribution to metastasis promotion is minimal and may be accounted for via their effects on the regulation of their oncotarget *c-Myc*.

## Materials and methods

### Patients and samples

We performed the experiments on the same 150 microsatellite-stable frozen matched mucosa and tumour samples with definitive metastasis status as previously reported^4^. Briefly, metastasis-positive case is defined as one with distal-organ involvement attributable to primary CRC; metastasis-negative case is defined as metastasis-free with 5 years or more follow-up. We excluded patients with microsatellite unstable tumours, because these are a small subset of sporadic CRCs with different biology^25^. We focused on left-sided (to the left of splenic flexture) tumours, as left and right-sided tumours are reported to have different biology^26^.

This study was approved by the SingHealth Centralized Institutional Review Board (2013/234/B). All research was performed in accordance with the relevant guidelines and regulations, and informed consent was taken from all participants and/or their legal guardians.

### Real-time RT-PCR assay

Taqman® real-time PCR analyses were performed on an Applied Biosystems™ 7900HT System using the FAM dye-labeled assay for target gene of interest pairing with primer-limited VIC dye-labeled assay for endogenous control (β-actin) in a single qPCR assay. The Taqman® expression assays are CCAT1 (Hs04402620_m1), CCAT2 (Hs04403001_s1) and MYC (Hs00153408_m1) for the targets and ACTB (Hs01060665_g1) for the endogenous control. cDNA from matched mucosa and tumour samples were run in quadruplicate on the same 384-well plate. The real-time PCR cycling conditions were: 50 °C 2 min, 95 °C 2 min, followed by 40 cycles of 95 °C 2 s and 60 °C 20 s. Relative expression of the 3 target genes in the tumours compared to matched mucosa was determined using the comparative Ct method (2-∆∆Ct).

### SNP genotyping assay

SNP genotyping was performed on DNA extracted from mucosa samples using the TaqMan® SNP Genotyping Assay (ThermoFisher Scientific, 4331349). Using the wet delivery method, 2.25 μL of DNA template was added to the reaction components according to the manufacturer’s instructions. The 384-well plate was run on an Applied BiosystemsTM 7900HT Real-Time PCR System at 95 °C for 10 min, followed by 40 cycles of 92 °C for 15 s and 60 °C for 1 min. Automatic allele calls were reviewed and converted into genotypes.

### Sanger sequencing

The primer sequences flanking the SNP rs698327 for PCR are 5′-GAGGGCACTAGACTGGGAAT and 5′-AAACTGAACTGTGGGGTTGG. The cycling conditions were: 95 °C for 2 min, followed by 28 cycles of 95 °C for 30 s, 57 °C for 45 s, and 72 °C for 30 s. The final extension step was run for 5 min at 72 °C. The purified PCR product underwent cycle sequencing using the BigDye Terminator v3.1 Cycle Sequencing Kit with primer sequence 5′-CCTGATTTCCCTTCCAGCTC. The cycling conditions were 96 °C for 1 min, followed by 25 cycles of 96 °C at 10 s, 50 °C for 5 s and 60 °C for 4 min. Sequencing product was precipitated then reconstituted in HiDi for sequencing using an Applied Biosystems™ 3500xL Genetic Analyzer.

### Statistical analysis

Statistical tests were performed using R 3.4.2 (https://www.r-project.org). Cox regression was used to evaluate the effect of different gene expression on time to metastasis. Time to metastasis is time from surgery to first clinical documentation of metastasis or if metastasis-free, to December 31, 2015. Kaplan–Meier analysis was used to evaluate the relationship between gene expression and time to metastasis. Mann–Whitney U or student’s t-test was carried out to compare gene expression levels between patients with different genotypes or with or without metastasis. Linear regressions were also carried out to evaluate the relationship of gene expressions between different genes. *p* values < 0.05 were considered statistically significant.

The Random Forest^27^ implementation from the Python machine learning package *scikit-learn*, v 0.17 was used as the machine-learning framework for the predictor^28^. Random Forest implementation uses training data to derive the “out-of-bag” (OOB) estimate of performance in new data which serves the same purpose as those obtained by cross validation^29^. Random Forests also rank features according to their importance in prediction^4^. ROC curves were performed to evaluate the performance of these genes to predict metastasis.

## Ethics approval and consent to participate

This study was approved by the SingHealth Centralized Institutional Review Board (2013/234/B). All research was performed in accordance with the relevant guidelines and regulations, and informed consent was taken from all participants and/or their legal guardians.

## Supplementary Information

Supplementary Figures.

## Data Availability

The data that support the findings of this study are available from the corresponding author upon reasonable request.

## Abbreviations

CRC: Colorectal cancer
*CCAT1*: Colon-cancer associated transcript 1
*CCAT2*: Colon-cancer associated transcript 2
HR: Hazard ratio
lncRNA: Long non-coding RNAs
NPV: Negative predictive value
OOB: “Out-of-bag”
PPV: Positive predictive value
ROC: Receiver operating characteristic
SNP: Single nucleotide polymorphism
TAD: Topologically associating domains
