# Supplementary material for: Enhancer-derived long non-coding RNAs CCAT1 and CCAT2 at rs6983267 has limited predictability for early stage colorectal carcinoma metastasis
Source: Sci Rep. 2021 Jan 11;11:404. doi: 10.1038/s41598-020-79906-7 (PMC7801656; doi:10.1038/s41598-020-79906-7)
Supplement: Supplementary file 1 — Supplementary Figures. [file 41598_2020_79906_MOESM1_ESM.docx]

**Supplementary Information**

Original Research

**Enhancer-derived** **long non-coding RNAs *CCAT1* and *CCAT2* has limited predictability for early stage colorectal carcinoma metastasis**

Lai Fun Thean ^1^, Christopher Blöcker ^2^, Hui Hua Li^3^, Michelle Lo^1^, Michelle Wong ^1^, Choong Leong Tang ^1^, Emile K. W. Tan ^1^, Steven G. Rozen^4^, Peh Yean Cheah^1,5,6^

^1^ Department of Colorectal Surgery, Singapore General Hospital, Singapore, Singapore

^2^ Department of Physics, Umeå University, 90187 Umeå, Sweden

^3^ Health Service Research Unit, Singapore General Hospital, Singapore, Singapore

^4^ Duke-NUS Center for Computational Biology, Duke-NUS Medical School, National University of Singapore

^5^ Saw Swee Hock School of Public Health, National University of Singapore, Singapore

^6^ Duke-NUS Medical School, National University of Singapore, Singapore

* Correspondence to:

Dr. Peh Yean Cheah, (ORCID 0000-0003-0403-8703)

Department of Colorectal Surgery, Singapore General Hospital, Academia, Level 9, Discovery Tower, 20 College Road, Singapore 169856

Email: [cheah.peh.yean@sgh.com.sg](mailto:cheah.peh.yean@sgh.com.sg) Tel.: (65) 63265365 Fax: (65) 62273787


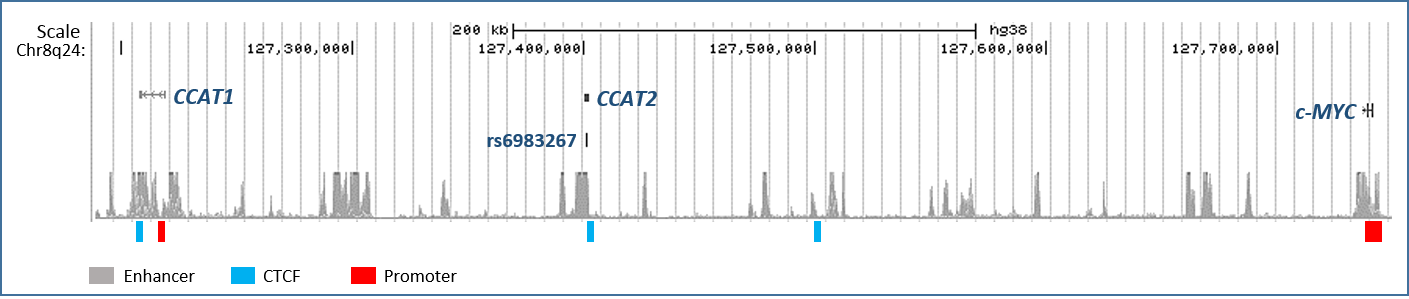


**Figure S1.** Schematic representation of physical position of *CCAT1*, *CCAT2* and *c-Myc* on chromosome 8q24.21.


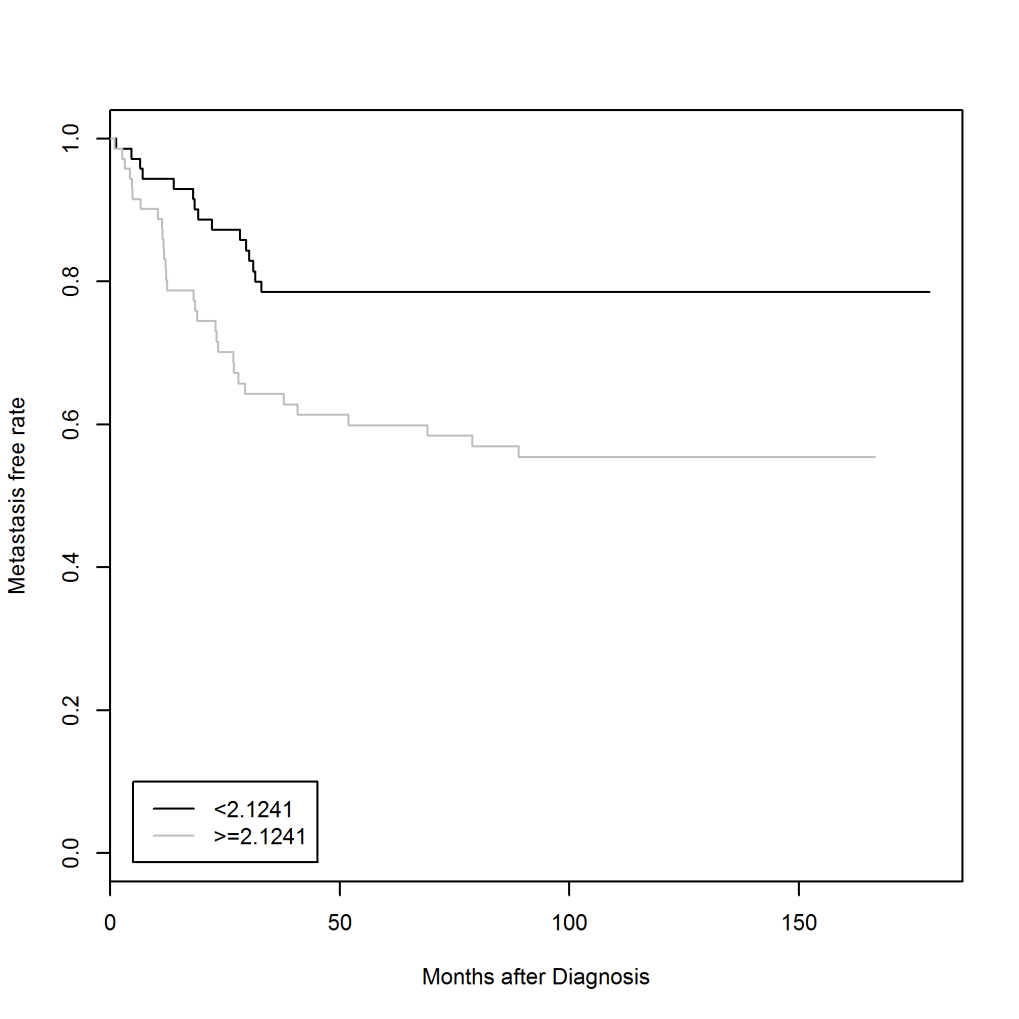


**Figure S2**. Kaplan-Meier plot of *c-Myc* High (>=2.1241) or Low (<2.1241) expression with Metastasis-free survival (Y-axis).
